# Spanish Translation, Cultural Adaptation and Validation of the Person‐Centred Practice Inventory‐Care (PCPI‐C): Enhancing Collaborative Care and Patient Involvement

**DOI:** 10.1111/jan.70542

**Published:** 2026-02-16

**Authors:** Ana Choperena, Ana Carvajal‐Valcárcel, Mónica Vázquez‐Calatayud, Juan Gamero‐Salinas, Begoña Errasti‐Ibarrondo, Virginia La Rosa‐Salas, Brendan McCormack, Vaibhav Tyagi, Edgar Benitez, Marta Lizarbe‐Chocarro

**Affiliations:** ^1^ Faculty of Nursing, ICCP‐UNAV Research Group‐Innovation for Person‐Centred Care University of Navarra Pamplona Spain; ^2^ The Navarra Institute for Health Research (IdiSNA) Pamplona Spain; ^3^ Clínica Universidad de Navarra Professional Development and Research Department Pamplona Spain; ^4^ Institute of Data Science and Artificial Intelligence (DATAI) University of Navarra Pamplona Spain; ^5^ TECNUN School of Engineering University of Navarra San Sebastián Spain; ^6^ Susan Wakil School of Nursing and Midwifery Sydney Australia; ^7^ Faculty of Medicine and Health The University of Sydney Sydney Australia; ^8^ Navarra Health Service—Osasunbidea University Hospital of Navarra (HUN) Pamplona Spain

**Keywords:** cross‐cultural adaptation, patients, person‐centred care (PCC), person‐centred practice (PCP), person‐centred practice inventory‐care (PCPI‐C), psychometric properties, validation

## Abstract

**Aim(s):**

To translate, culturally adapt and validate the first Spanish version of the Person‐centred Practice Inventory‐Care (PCPI‐C) instrument.

**Design:**

Cross‐cultural adaptation and psychometric validation.

**Methods:**

Two‐phase research design: (1) the PCPI‐C's translation and cultural adaptation from English to Spanish following the ‘Translation and Cultural Adaptation of Patient‐Reported Outcomes Measures‐Principles Guide of Good Practice’ tool; and (2) a cross‐sectional quantitative survey to assess the Spanish version's psychometric properties.

**Results:**

A sample of 200 patients participated to obtain the PCPI‐C's Spanish version. No significant issues arose during the translation process or the consulting sessions. No item exhibited an inadequate value following adjustment via the weighted kappa index (−scale‐level content validity average of 0.95 for clarity and 0.97 for relevance). Psychometric evaluation revealed acceptable internal consistency (Cronbach's alpha from 0.67 to 0.84) and strong construct validity. Exploratory and confirmatory factor analyses supported a five‐dimensional structure consistent with the domain Person‐Centred Processes. Fit indices improved after model refinements, achieving CFI = 0.92, SRMR = 0.05 and RMSEA = 0.07. This study's observed psychometric properties confirm that the PCPI‐C's Spanish version retains the original instrument's theoretical integrity, while showing strong reliability and validity in the new context.

**Conclusion:**

The PCPI‐C's Spanish translation was psychometrically valid when tested with Spanish patients, thus providing a culturally appropriate, psychometrically sound tool to evaluate Spanish‐speaking patients' perception of person‐centred care.

**Impact:**

This study provides a validated instrument that allows for the assessment of person‐centred practice in Spanish‐speaking clinical environments. It enables healthcare professionals to measure patients' perceptions, track the implementation of person‐centred principles and supports international comparative studies, contributing to the development of more ethical and responsive models of care.

**Patient or Public Contribution:**

Patients participated in cognitive consultations and completed the survey for psychometric testing, ensuring that the translated items were understandable, culturally appropriate and reflective of their experiences of person‐centred care.

## Key Points

- What problem did the study address?
  ○ Inconsistencies in understanding and operationalising Person‐Centred Practice (PCP) across healthcare contexts have been reported.
  ○ There is a need to evaluate, translate and adapt PCP in culturally diverse settings.
- What were the main findings?
  ○ A valid, reliable Spanish version of the PCPI‐C was obtained.
  ○ The PCPI‐C enables identification of patients' perceptions of person‐centred practice, tracking this practice's evolution over time and assessing interventions to improve person‐centred practice.
- Where and whom will the research impact?
  ○ The results presented in this article will enable international comparative studies and support PCP's further development in Spanish‐speaking clinical settings.
  ○ Having instruments aligned with a theoretical person‐centred framework enables healthcare professionals and patients to use a standardised measure to evaluate the degree to which person‐centred principles have been implemented.

## 1 Introduction

Person‐centred care (PCC) represents a paradigm shift in contemporary healthcare, moving beyond efficiency‐driven models towards a more humanised and individualised approach (McCance and McCormack 2025; McCormack et al. 2015). PCC places the individual's beliefs, values and preferences at the centre of clinical decision‐making, fostering conditions for healthcare professionals to engage with patients who are active partners in their care (McCance et al. 2021; Pushparajah 2018). Distinct from traditional models of shared decision‐making, PCC emphasises respectful, collaborative relationships and development of tailored interventions that respond to individuals' specific contexts and needs (McCance et al. 2021; McCormack et al. 2021).

From there, McCormack and McCance developed the Person‐centred Practice Framework (PCPF), an internationally recognised model, focused on person centred practice (PCP), that provides a comprehensive, theoretically grounded approach to implement the PCC approach (McCance et al. 2021; McCormack 2024; McCormack et al. 2021). Originating in the nursing field (McCormack and McCance 2016; McCance and McCormack 2025), the PCPF has evolved into a transdisciplinary framework with applicability across diverse healthcare settings. Through two decades of development, the framework has contributed significantly to person‐centredness's advancement by offering a shared language and a coherent structure for understanding its practical implementation (McCormack 2024; McCormack et al. 2021).

**FIGURE 1:**
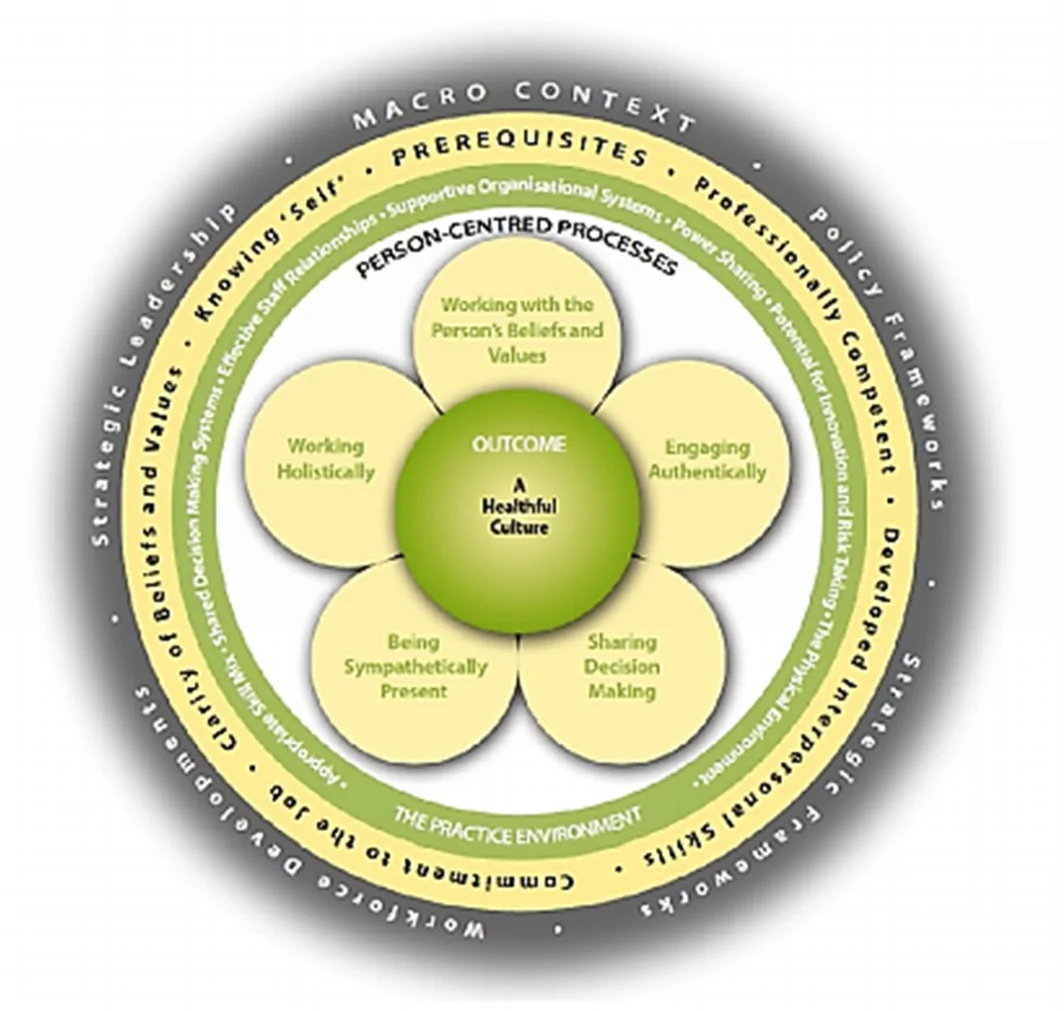
Person centred practice framework (PCPF) by McCance et al. (2021).

The PCPF consists of five interrelated domains (Figure 1): *prerequisites*, encompassing healthcare providers' personal and professional attributes; *the care environment*, referring to the organisational context in which care is delivered; *person‐centred processes*, describing how meaningful engagement with patients is achieved; *person‐centred outcomes*, representing such engagement's effects; *the macro context*, including broad strategic and policy‐level influences (McCormack et al. 2021). At the framework's centre lies the concept of a healthful culture—an organisational climate that supports both high‐quality care and healthcare staff's wellbeing. This culture cannot be achieved without attending to all the framework's levels, from individual competencies to system‐wide structures and values (McCance et al. 2021; McCormack et al. 2021).

A key element in advancing PCP is the availability of valid, reliable instruments that measure its implementation and impact. In recent years, several instruments have been developed to assess experiences—Patient‐Reported Experience Measures (PREMs)—and their perceived health outcomes, or Patient‐Reported Outcome Measures (PROMs) (Havana et al. 2023; Kidanemariam et al. 2024; Lloyd et al. 2019). Although these instruments offer valuable insights, most were designed from a single perspective, predominantly that of the patient and often lack alignment with a consistent theoretical framework. Furthermore, few studies have incorporated the complexity of care environments or the quality of inter‐professional relationships that shape person‐centred practice (Fridberg et al. 2020; Lloyd et al. 2019; Rodríguez‐Martín et al. 2019).

Although such instruments as the Person‐Centred Coordinated Care Experience Questionnaire (P3CEQ) (Lloyd et al. 2019), the Integrated Care Survey (Rodríguez‐Martín et al. 2019) and the Gothenburg Person‐Centred Care Questionnaire (Fridberg et al. 2020) provide valuable insights into patient experiences, they often overlook relational and contextual dimensions fundamental to PCP as conceptualised in the PCPF (Handley et al. 2021). To address these gaps, Slater et al. (2017) developed the Person‐Centred Practice Inventory‐Staff (PCPI‐S), a theoretically grounded tool that assesses healthcare professionals' perceptions across key PCPF domains (Slater et al. 2017). Its widespread use and strong psychometric properties have been demonstrated (Carvajal‐Valcárcel et al. 2024). Similarly, to evaluate patients' perspectives from the same framework approach, the Person‐centred Practice Inventory‐Care (PCPI‐C) focuses on individuals' experiences of person‐centred processes such as authentic engagement, shared decision‐making and holistic care (McCormack et al. 2024, 2025). The PCPI‐C complements the PCPI‐S, allowing for comprehensive, dual‐perspective assessment of PCP.

In our globalised world, where major health challenges demand prioritisation of individuals at healthcare practice's centre, PCP's evaluation across diverse cultural settings is crucial. Translating and culturally adapting instruments such as the PCPI‐C—as has been done for French (Mabire et al. 2024), Danish (Rosted et al. 2025) and Portuguese populations (Vareta et al. 2025)—is a vital step in Spanish‐speaking contexts as well. Therefore, this study developed the PCPI‐C's first Spanish version, ensuring its translation, cultural adaptation and validation for use in Spain, thus enabling assessment of person‐centred practices via a shared, theoretically grounded framework.

## 2 Method

Given the background above, this research completed cultural adaptation and psychometric testing of a Spanish PCPI‐C iteration (McCormack et al. 2024). A two‐phase research design was employed: (1) the PCPI‐C's translation and cultural adaptation from English to Spanish using the nominal group technique and (2) a cross‐sectional quantitative survey to assess the adapted version's psychometric properties.

### 2.1 Instrument Overview

The PCPI‐C is an 18‐item instrument designed to assess how person‐centredness is perceived by service users across varied healthcare contexts and organisational levels. It comprises five subscales (constructs) aligned with the domain *Person‐centred processes* domain from the PCPF: *Working with the person's beliefs and values*, *Sharing decision‐making*, *Engaging authentically*, *Being sympathetically present* and *Working holistically*. Each item is rated on a 5‐point Likert scale ranging from strongly disagree (1) to strongly agree (5), without reverse scoring items. The original version's Cronbach alpha scores ranged from 0.55 to 0.76 across constructs, indicating acceptable internal consistency and supporting each construct's one‐dimensionality. Confirmatory factor analysis, with a 90% root mean square estimation of approximation (among others) from 0.045 to 0.06, demonstrated that the PCPI‐C is psychometrically sound, supporting a five‐factor model (McCormack et al. 2024, 2025).

### 2.2 Phase 1: Translation and Cultural Adaptation

#### 2.2.1 Procedure Overview

The translation process followed the ten‐step ‘Translation and Cultural Adaptation of Patient‐Reported Outcomes Measures‐Principles of Good Practice Guide’ (Wild et al. 2005).

**FIGURE 2:**
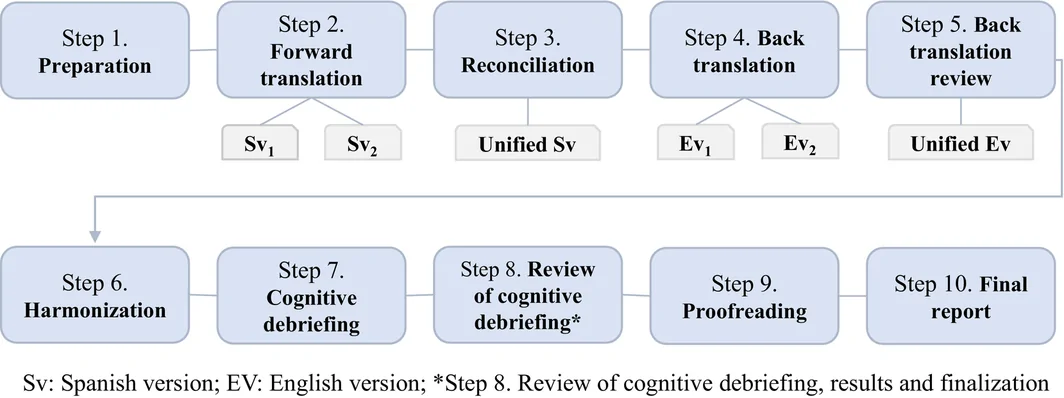
‘Translation and Cultural Adaptation of Patient‐Reported Outcome Measures—Principles of Good Practice’ (Wild et al. 2005). EV, English version; Sv, Spanish version; *Step 8. Review of the cognitive debriefing results and finalisation.

Figure 2 illustrates and details this process, including: Step 1: *Preparation*; Step 2: *Forward translation*; Step 3: *Reconciliation*; Step 4: *Back translation*; Step 5: *Back translation review*; Step 6: *Harmonisation*; Step 7: *Cognitive debriefing* during which a group of PCP experts and service users rated the clarity and relevance of the Unified Spanish Version (SV_u_) using a Likert scale ranging from 1 (*not clear or relevant*) to 4 (*very clear or relevant*). For items scored 1 (*not*) or 2 (*slightly*) on relevance or clarity, an audio‐recorded group discussion by participants explained and elaborated reasons for the scores, provided suggestions for improvement or alternative wording, and justified any discrepancies. Subsequently, a rigorous critical review of the cultural relevance of each item was undertaken. Each item was meticulously assessed for its appropriateness, comprehensibility and cultural congruence within the specific Spanish context. This process specifically involved evaluating whether particular concepts, wording or illustrative examples embedded within the items might elicit differential interpretations or be perceived as culturally offensive or irrelevant by the target population (Beaton et al. 2000; Wild et al. 2005). In Step 8, *Review of cognitive debriefing* results and *finalisation*, content validity analyses (for each item and the entire scale) were calculated. Specifically, the item‐level content validity index (I‐CVI) and the scale‐level content validity index average (S‐CVI/Ave) were assessed. The I‐CVI was calculated by dividing the number of observers scoring items with 3 (*clear or relevant*) or 4 (*very clear or relevant*) by the total number of observers. Additionally, the modified kappa statistic was calculated to adjust each I‐CVI to minimise random agreement between experts (Polit et al. 2007). Interpretation of kappa values followed the Cicchetti and Sparrow (1981) classification. The content validity of the entire PCPI‐C Spanish version (S‐CVI/Ave) was calculated as the mean of all I‐CVIs and interpreted according to Waltz et al. (2010) (> *o* = 0.90 excellent). Comments from experts and service users were examined through content analysis. Analyses of comments from the cognitive debriefing were considered. The PCPI‐C's original lead author was consulted to resolve any discrepancies. Step 9: *Proofreading*; and Step 10: *Final report*. At each stage, participants' time to complete the task was recorded.

#### 2.2.2 Translation and Adaptation Working Group Characteristics

Participants in the different phases of the study's Phase I process included: (1) one author of the PCPF and the lead author of the PCPI‐C; (2) the principal researcher (a native Spanish speaker fluent in English, residing in Spain, with a background in health sciences and experience translating instruments); (3) two forward translators (both native Spanish speakers fluent in English, one of whom is a professional translator with expertise in healthcare contexts and PCP); (4) two back translators (both native English speakers and professional translators fluent in Spanish, who were not familiar with PCP or health sciences); (5) a conciliation committee comprised of the two forward translators, two researchers and the principal researcher; (6) a PCP expert panel conveniently selected, that is, six specialists with diverse backgrounds in: (a) academic training (lecturers and professors), (b) experience in instrument validation (one expert) and (c) professional roles (clinical practice, combined clinical and academic roles and healthcare management); (7) a group of service users (two males, three females, mean age = 64.8 [SD = 12.7]), recruited by convenience from an outpatient setting; and (8) an expert in humanities with previous experience in grammatical and linguistic editing.

#### 2.2.3 Data Collection

The initial research phase—the PCPI‐C's translation and cultural adaptation into Spanish—was conducted from March to December 2022. All eligible persons involved in the translation and cultural adaptation—both expert panellists and patient service users—received comprehensive verbal and written information regarding their involvement's voluntary nature, their data's strict confidentiality and anonymity, and the data's exclusive use for scientific purposes.

### 2.3 Pilot Test

After completion of the translation and cross‐cultural adaptation process and prior to psychometric validation, a pilot test was conducted with seven outpatients (three males, four females with a mean age of 68.2, Standard deviation [SD] = 13.2) from the target population to further refine the instrument's cultural adaptation and linguistic appropriateness (Wild et al. 2005). The pilot test assessed the adapted instrument's readability and understandability to identify any remaining ambiguities, difficulties in comprehension or unintended cultural nuances that may not have emerged during the expert panel consensus. Patients completed the PCPI‐C's Spanish version via face‐to‐face administration. The time taken to complete the questionnaire was recorded, and any comments on clarity or wording were noted.

### 2.4 Phase 2: Psychometric Evaluation

#### 2.4.1 Context and Participants

The study was conducted at Blinded for review, a highly specialised tertiary university hospital in Spain with Magnet designation for nursing excellence. The institution's nursing practice is framed by the Professional Nursing Practice Model (Rumeu‐Casares et al. 2017) that emphasises person‐ and family‐centred care, clinical leadership, professional autonomy, interdisciplinary collaboration and research. This model aligns with the PCPF, which underpins the instrument culturally adapted, translated and validated in this study.

Convenience sampling was used to recruit participants from both inpatient and outpatient settings, including medical, surgical and medical‐surgical units. Inclusion criteria for hospitalised patients were (1) adults aged 18 or over, (2) hospitalised for at least 3 days, (3) with normal consciousness and a clinical condition permitting questionnaire completion, (4) Spanish language proficiency and (5) provision of written informed consent. For outpatients, criteria included (1) adults aged 18 or over, (2) attending outpatient clinics for the specialties mentioned above, (3) with Spanish language proficiency and (4) willingness to provide written informed consent.

Prior to recruitment, institutional approval was obtained, and nurse managers from relevant units were briefed; they acted as gatekeepers by identifying eligible patients and facilitating contact with the research team. Researchers without direct clinical responsibility for patients conducted the recruitment in person. Inpatients were approached on their third day of hospitalisation; outpatients were invited to participate during their pre‐consultation wait time. This personalised, face‐to‐face recruitment strategy, supported by dedicated research staff, is recognised as effective in maximising participation rates and minimising selection bias (Preston et al. 2016). All participants provided written informed consent prior to participation.

#### 2.4.2 Recruitment and Data Collection

Participants were systematically identified in both outpatient and inpatient settings. A consecutive sampling approach was used.

In the outpatient setting, researchers screened daily appointment lists supplied by administrative staff to identify potentially eligible patients. All eligible patients attending consultations during the data collection period were invited to participate until the required sample size was reached. Outpatients completed the questionnaire while waiting for their scheduled consultation, with researchers available to provide support if required. In the inpatient setting, the research team reviewed the daily hospital census in collaboration with ward nursing staff. Patients hospitalised for at least 3 days and meeting the inclusion criteria were identified and approached in their rooms. This process ensured comprehensive and equitable identification of eligible inpatients. Hospitalised patients received the self‐reported questionnaire on the morning of their third day of admission. The same researcher collected it later that afternoon, allowing sufficient time for completion and addressing questions when needed.

All eligible individuals received verbal and written information explaining the voluntary nature of participation, confidentiality and anonymity safeguards and the scientific purpose of data use. Participants were explained that the aim of the study was to explore their perceptions of how they had been cared for by healthcare professionals. They were invited to think about the last time they had been attended to by health professionals (e.g., nurses, physicians, nursing assistants) and to respond to the questionnaire based on that experience.

All participants provided basic demographic information (gender, age and educational level) before completing the questionnaire. The primary data collection method employed was a self‐completed questionnaire to ensure anonymity and minimise interviewer bias. Data were collected between October and December 2023 by research team members with no clinical involvement in patient care, thereby reducing the risk of bias.

#### 2.4.3 Sample Size

Based on statistical guidelines (Maccallum et al. 1996), the required sample size to achieve statistical power of 80% was calculated. The analysis used the following input parameters: *α* = 0.05 (level of significance), root mean square error of approximation (RMSEA) (null hypothesis) = 0.05 (close fit under the null hypothesis), RMSEA (alternative hypothesis) = 0.08 (adequate fit under the alternative hypothesis) and degrees of freedom (df) = 1364. Results indicated that a minimum sample size of 200 was necessary to meet the desired level of statistical power. A post hoc power analysis was conducted using the R package *semPower* (Moshagen and Bader 2024) to assess whether the sample size (*N* = 200) was sufficient to detect the observed level of model misfit based on RMSEA from CFA.

#### 2.4.4 Data Analysis

Descriptive statistics were calculated for all items. Preliminary analyses included Cronbach's alpha to assess internal consistency before conducting CFA and the Kaiser–Meyer–Olkin (KMO) measure and Bartlett's test of sphericity to evaluate the data's suitability for factor analysis. Additionally, data normality was examined using skewness and kurtosis, with values considered acceptable in the range of −1.5 to +1.5 (Lloret‐Segura et al. 2014). For CFA, all items were rank‐transformed, with each value replaced by its ordinal position within data distribution (a nonlinear transformation) to preserve observations' relative ordering and to treat the items as continuous rather than ordinal variables. By preserving the robust rank‐ordering while normalising the data, this approach leverages the benefits of parametric procedures on non‐normal data, serving as a methodological bridge between parametric and nonparametric statistics (Conover and Iman 1981).

As suggested by Anderson and Gerbing (1988), CFA was conducted using the simplest model of fit and the *lavaan* package (Rosseel 2012) in Rstudio. All available observations (*n* = 200) were used; missing values were treated using the argument *missing = ‘fiml’* (full information maximum likelihood), which allows maximising available information's use without prior imputation. To address potential violations of multivariate normality, CFA was estimated using the robust maximum likelihood estimator (MLR), which provides Huber–White standard errors and a scaled test statistic adjusted for non‐normality (Lloret‐Segura et al. 2014).

Model fit was evaluated using the following criteria: RMSEA of 0.05 or below; 90% RMSEA upper limit below 0.08; confirmation fit indices (CFI) of 0.95 or above; standardised root mean square residual (SRMR) below 0.05. Modifications, guided by Ward's test and Lagrange multipliers, were applied sequentially if theoretically justified, and if modification indices (MI) were above 0.25, to maximise model parsimony as conducted in the original 18‐item PCPI‐C theoretical model (McCormack et al. 2024).

Construct reliability and validity were assessed by calculating Composite Reliability (CR) and Average Variance Extracted (AVE) from the standardised factor loadings of the final CFA model (Fornell and Larcker 1981). CR was used to assess construct reliability, with values of > 0.70 indicating adequate internal consistency. AVE was used to assess convergent validity, with a criterion of > 0.50 indicating that the factor explained more than half of the variance in its items. Additionally, item reliability was assessed, with values > 0.40 considered satisfactory (O'Rourke and Hatcher 2013).

### 2.5 Ethical Approval

The study was approved by the Clinical Research Ethics Committee (CEIC) of the University of Navarra (Approval No. 2021.125 Mod1). Authorisation to use the PCPI‐C was provided by its original authors.

## 3 Results

### 3.1 Phase 1: Translation and Cultural Adjustment

#### 3.1.1 Step 1. Preparation

Authorisation to translate the instrument was obtained from the lead author of the PCPI‐Care (BMc) (representing the co‐authors) who also agreed to be involved in the proceedings.

#### 3.1.2 Step 2. Forward Translation

Following the translation of the instrument from English to Spanish, Spanish versions (SV) one (Sv_1_) and two (Sv_2_) were obtained. The average time spent on the process was half an hour. Both translators reported the translation straightforward and the language clear.

#### 3.1.3 Step 3. Reconciliation

The conciliation committee of researchers thoroughly reviewed Sv_1_ and Sv_2_ without finding significant discrepancies. They concluded the two were similar, so no difficulties arose reaching consensus on any of the questionnaire's 18 items and agreeing on the unified Spanish version (SV_u_).

#### 3.1.4 Step 4. Back Translation

Two independent conceptual back translations into English (Ev_1_ and Ev_2_) from the SV_u_ focused on preserving conceptual meaning rather than literal translation. The translation process for the questionnaire required 20 min and back translators reported no difficulties.

#### 3.1.5 Step 5. Back Translation Review

Without difficulties, the reconciliation committee reviewed Ev_1_ and Ev_2_ to produce a unified English version (Ev_u_). This version was compared with the original one by the original author (BMc), who suggested two clarifications: (1) in Item 4, retaining ‘home environment’ instead of ‘family environment’ to preserve broader meaning, and (2) in Item 8, revising the translation of ‘staff ask me about’ to ‘health staff show interest’ the better to reflect the original's intent. Therefore, the Sv_u_ was amended, with Item 8 translated as ‘El personal se interesa por mi vida’ (‘The staff are interested in my life’) for greater conceptual accuracy.

#### 3.1.6 Step 6. Harmonisation

Harmonisation was deemed unnecessary because during the study, no other PCPI‐C language versions were available.

#### 3.1.7 Step 7. Cognitive Debriefing

On the PCPI‐C's Spanish version, the PCP expert panel and service user group averaged a completion time of 4 min. Subsequently, a cognitive debriefing session of approximately 2 h was conducted. Items receiving low scores (1 or 2) for clarity and relevance were discussed, and alternatives were suggested. All participants also reflected on the questionnaire's general clarity and the self‐perception it prompted.

#### 3.1.8 Step 8. Review of the Cognitive Debriefing Results and Finalisation

For clarity, 16 of the 18 items achieved an excellent item content validity index (I‐CVI), and the scale content validity index/average (S‐CVI/Ave) was 0.95. For relevance, all items reached an excellent I‐CVI, and the S‐CVI/Ave was 0.97 (Table 1). After content analysis, the expert and user panel's proposals and suggestions were accepted or rejected according to the questionnaire author and the principal investigator's instructions (Table 2). Of all the points made to assure cultural testing, the main one was Item 14 ‘Staff respond compassionately when I am upset or unhappy’: experts unanimously agreed that the text include its definition due to its different connotations in Spanish culture. Besides that, one university‐educated service user said the questionnaire was clear and relevant overall, but was unsure whether users at varied cultural levels could complete it. Furthermore, four service users emphasised that, according to its instructions, the instrument reflects users' perception of how they were cared for *the last time* rather than *over time*.

**TABLE 1:** Clarity and relevance of the Person‐Centred Practice Inventory‐Care (PCPI‐C)–Spanish version.

Person‐centred process domains		Clarity			Relevance		
		I‐CVI	K*	Evaluation	I‐CVI	K*	Evaluation
	**Working with the Person's Belief and Values**						
Item1	Staff make an effort to understand what is important to me.	1	1	E	0.91	0.91	E
Item 6	I feel able to say to staff what is important to me.	0.91	0.91	E	1	1	E
Item7	I feel able to give staff feedback about my experience of being cared for.	0.73	0.70	G	1	1	E
Item 12	In caring for me, the staff use what they know about me as a person.	0.91	0.91	E	0.91	0.91	E
	**Sharing Decision‐making**						
Item 3	The staff involved me in making decisions about my care.	1	1	E	1	1	E
Item 10	Staff ask me if I have all the information I need.	1	1	E	1	1	E
Item 15	The staff helped me to express my concerns about my treatment and care.	1	1	E	1	1	E
Item 18	Staff help me to set realistic goals.	1	1	E	1	1	E
	**Engaging Authentically**						
Item 9	Staff connect with me as a person.	0.91	0.91	E	1	1	E
Item 11	When we disagree about my care, the staff try to find common ground.	1	1	E	1	1	E
Item 16	Staff listen to me and hear what I have to say about my care.	1	1	E	0.91	0.91	E
	**Being Sympathetically Present**						
Item 2	Staff use my personal experiences to build a relationship with me.	0.73	0.70	G	1	1	E
Item 5	Staff give me their full attention when they are with me.	1	1	E	1	1	E
Item 14	Staff respond compassionately^a^ when I am upset or unhappy.	1	1	E	1	1	E
	**Working Holistically**						
Item 4	Staff consider my home environment in meeting my care needs.	1	1	E	1	1	E
Item 8	Staff ask me about my life.	0.82	0.81	E	1	1	E
Item 13	I feel cared for.	1	1	E	1	1	E
Item 17	Staff understand my family circumstances when caring for me.	1	1	E	0.91	0.91	E
		S‐CVI/Ave = 0.95			S‐CVI/Ave = 0.97		

*Note:* Evaluation criteria for kappa, using guidelines described in Cicchetti and Sparrow (1981) and Fleiss (1971): Fair (F) = *K* of 40 to 59; Good (G) = *K* of 60 to 74; and Excellent (E) = *k* > 0.74.

Abbreviations: I‐CVI, item‐level content validity index; K*, Kappa designating agreement on relevance; S‐CVI/Ave, Scale‐level content validity/average according to Waltz & col (2005) (Excellent ≥ 0.90).

^a^ Compassion is the sensitivity shown by the healthcare professional to understand the suffering of another person, combined with the willingness to help and promote wellbeing in order to find a solution to the other's problem (Perez‐Bret et al. 2016).

**TABLE 2:** Step 8: Review the cognitive debriefing results and finalisation.

Original question	Suggestions provided	WHO suggest	Final decision
*Item 2. El personal tiene en cuenta mi experiencia personal para establecer una relación conmigo*	Add personal experience, desires, values, beliefs, preferences, etc.	1 patient	Modification was not deemed necessary because only one individual suggested the change, and it was already implicit in the phrasing.
*Item 3. El personal me involucra en la toma de decisiones acerca de mi cuidado*	Clarify the decision‐making process by adding in parentheses: taking into account my opinion, preferences, etc.	1 patient	Further clarification was not contemplated due to its perceived redundancy.
*Item 5. El personal me dedica toda su atención cuando recibo su cuidado*	To clarify, *they provide their complete attention to me*, as evidenced by their gaze, attentiveness and expressed interest in my current state.	1 patient	It was not deemed pertinent because it was already implicit in the phrasing.
*Item 13. Me siento cuidado/a*	Add ‘well’ to the sentence: *Me siento bien cuidado/a*.	2 Experts	The questionnaire author did not consider it appropriate because adding ‘well’ was a value judgement, and stating ‘I feel cared for’ allowed for varying degrees of perceived care.
*Item 14. Cuando me siento disgustado o triste, el personal sanitario me cuida de forma compasiva*	Clarify the concept of ‘compassion’	All PCP expert groups	Due to its varying connotations in Spanish, it was unanimously decided to include its definition.

*Note:* Items 1, 2, 3, 4, 5, 8, 9, 10, 12, 15, 16, 17 and 18. All items begin with the phrase *El personal sanitario*; therefore, it was suggested to use *El personal sanitario* in the first item and, from the second item onwards, to employ the word *personal* to avoid redundancy and prevent patients from responding mechanically to the questionnaire.

#### 3.1.9 Step 9. Proofreading

The humanities expert identified no linguistic errors in the final Spanish version. She suggested a minor modification to Item 13 (‘My family is involved in decisions about my care as long as I agree’) to enhance the linguistic style. However, to maintain maximum fidelity to the original English version, this proposed change was not implemented.

#### 3.1.10 Step 10. Final Report

The Spanish PCPI‐C's final version was achieved when the main researcher compiled the final report, outlining the research process and methodology (Table S1).

### 3.2 Pilot Test

A pilot test was conducted with seven patients, who spent an average of 4.3 min (between 4 and 5) completing the instrument. All commented that it was easy to understand and complete, and all rated it as simple. The majority (85.7%) highlighted its clarity and conciseness, and all (100%) indicated it was short, simple and easy‐to‐complete. No significant cultural divergences were identified. Furthermore, they agreed that the questionnaire's items captured what PCP (likely referring to a specific concept being studied) meant to them and suggested no additional items.

### 3.3 Phase 2: Psychometric Evaluation

Data collection with patients was conducted over a three‐month period. All potential participants invited to take part in the research completed the questionnaire, resulting in a 100% response rate. Service users (56.5% female; mean age = 58.5 [standard deviation = 15.9]; age range 16–93) completed 200 questionnaires, with 8 missing some values. Table 3 details these participants' demographic characteristics.

**TABLE 3:** Sample characteristics.

	*n*	Percentage (%)
**Gender**		
Female	113	56.5
Male	85	42.5
Not specified	2	1.0
**Educational Level**		
Primary (0–8 years)	10	5.0
Medium (8–12 years)	48	24.0
Higher (> 12 years)	138	69.0
Not specified	4	2.0
**Care Setting**		
Outpatient^a^	93	46.5
Inpatient^b^	107	53.5

^a^ Breast pathology consultation (*n* = 7); Neurosurgery consultation (*n* = 9); Cardiology consultation (*n* = 9); Ophthalmology consultation (*n* = 1); Allergology consultation (*n* = 9); Pain Management Unit (*n* = 4).

^b^ Oncology (*n* = 10); Orthopaedic Surgery (*n* = 7); General Surgery (*n* = 5); Cardiology (*n* = 12); Internal Medicine (*n* = 7); Intensive Care Unit (*n* = 2).

No item showed a skewed distribution outside the [−1.5, +1.5] range, but items 1, 3, 6, 10, 12, 16 and 17 showed kurtosis values outside that range, so they were log‐transformed for subsequent analyses. Item 1 retained a kurtosis value below −1.5 even after log‐transformation. Once items were rank‐transformed, the KMO (0.93) and Bartlett's test of sphericity (2761.95, df = 153, *p* < 0.001) indicated suitability for factor analysis. Cronbach's alpha scores for each construct confirmed internal consistency, with only Factor 4 falling slightly below the commonly accepted threshold of 0.70. Table 4 summarises descriptive statistics and factor loadings.

**TABLE 4:** Descriptive statistics and factor loadings for PCPI‐C–Spanish version.

Person‐centred processes	Item No.	Mean	Std.	Skewness	Kurtosis	F. loading
**Working with the Person's Belief and Values (*α* = 0.83)**						
Staff make an effort to understand what is important to me.	1*	4.54	0.55	−0.31	−1.80	0.79
I feel able to say to staff what is important to me.	6	4.41	0.65	−0.17	−1.61	0.62
I feel able to give staff feedback about my experience of being cared for.	7*	4.24	0.75	−0.11	−1.35	0.67
In caring for me, the staff use what they know about me as a person.	12*	4.48	0.63	−0.32	−1.65	0.82
**Sharing Decision‐making (*α* = 0.81)**						
The staff involved me in making decisions about my care.	3*	4.44	0.63	−0.24	−1.60	0.71
Staff ask me if I have all the information I need.	10*	4.54	0.58	−0.46	−1.62	0.74
The staff helped me to express my concerns about my treatment and care.	15	4.30	0.72	−0.07	−1.41	0.81
Staff help me to set realistic goals.	18	4.35	0.64	−0.03	−1.48	0.79
**Engaging Authentically (*α* = 0.77)**						
Staff connect with me as a person.	9	4.30	0.71	−0.13	−1.42	0.72
When we disagree about my care, the staff try to find common ground.	11	4.32	0.72	−0.22	−1.48	0.75
Staff listen to me and hear what I have to say about my care.*	16*	4.51	0.59	−0.33	−1.70	0.81
**Being Sympathetically Present (*α* = 0.67)**						
Staff use my personal experiences to build a relationship with me.	2	4.35	0.70	−0.19	−1.50	0.69
Staff give me their full attention when they are with me.	5	4.66	0.49	−0.76	−1.40	0.73
Staff respond compassionately when I am upset or unhappy.	14	4.17	0.72	−0.05	−1.24	0.66
**Working Holistically (*α* = 0.84)**						
Staff consider my home environment in meeting my care needs.	4	4.31	0.74	−0.21	−1.46	0.75
Staff ask me about my life.	8	4.33	0.68	−0.16	−1.47	0.76
I feel cared for.	13	4.68	0.52	−0.90	−1.19	0.73
Staff understand my family circumstances when caring for me.	17*	4.38	0.69	−0.23	−1.52	0.79

*Note:* Cronbach's alpha (*α*), means and standard deviations (Std.) shown are before rank‐transformation. Items with * were log‐transformed. All factor loadings from CFA have *p* > 0.001.

To evaluate the instrument's structural validity, CFA was conducted using maximum likelihood estimation with robust correction (MLR) on both the original theoretical model and a fitted model based on the modification indices (Table 5). Nine further modifications were introduced into the original model based on only residual correlations with MI > 0.25 (explained in the Methods section). Although the literature supports thresholds as low as 0.11 (Vareta et al. 2025), this study avoided overfitting due to excessive parameter addition by incorporating only theoretically justified residual covariances with high MI values. Of these, one correlated error modification occurred in a single factor (*Working with the Person's Beliefs and Values*: Items 6 and 7), while the remaining eight involved correlated errors between factors. Only one correlated error was negative (between Items 9 and 4).

**TABLE 5:** Fit statistics for the PCPI‐C–Spanish model (before and after model re‐specification).

Model	Chi‐squared (*χ* ^2^)	*p*	df	CFI	SRMR	RMSEA	90% RMSEA
Original Model (five factors)	363.84	*p* < 0.001	125	0.844	0.064	0.098	0.089–0.106
Fitted Model (with MI)	232.18	*p* < 0.001	116	0.924	0.049	0.071	0.061–0.081

Abbreviations: 90% RMSEA, Root Mean Square Error of Approximation Confidence Level; CFI, Comparative Fit Index; df, degrees of freedom; RMSEA, Root Mean Square Error of Approximation; SRMR, Standardised Root Mean Square Residual.

The fitted CFA model (with MI) results show acceptable factor loadings, with values ranging from 0.618 to 0.823. Model fit indices were acceptable, with an RMSEA of 0.071 (90% CI [0.061, 0.081], SRMR = 0.049, CFI = 0.924 and TLI = 0.900). Although RMSEA exceeded the conventional cut‐off of 0.05, it remained within acceptable range (< 0.08), especially given relatively high CFI and SRMR below 0.05.

According to post hoc power analysis, the sample size of 200 participants yielded high statistical power (0.99995) to detect the misfit level represented by RMSEA = 0.071. Comparatively, power remained acceptable (0.9359) for an RMSEA of 0.05, confirming the sample's adequate size.

To assess reliability and convergent validity, construct/item reliability (CR) and average variance extracted (AVE) were calculated for each factor (Table 6). All constructs except *Being sympathetically present* met the recommended AVE threshold (> 0.49), and all CR values exceeded the 0.70 criterion. Except for Item 6 (‘I feel able to say to staff what is important to me’) with a value of 0.381, most items exhibited high individual reliability (> 0.39) that supported internal consistency.

**TABLE 6:** Reliability and convergent validity of the PCPI‐C–Spanish version.

Item	Construct	Reliability	AVE
	**Working with the Person's Belief and Values**	0.817	0.530
1	Staff make an effort to understand what is important to me.	0.620	
6	I feel able to say to staff what is important to me.	0.381	
7	I feel able to give staff feedback about my experience of being cared for.	0.442	
12	In caring for me, the staff use what they know about me as a person.	0.677	
	**Sharing Decision‐making**	0.845	0.578
3	The staff involved me in making decisions about my care.	0.502	
10	Staff ask me if I have all the information I need.	0.541	
15	The staff helped me to express my concerns about my treatment and care.	0.650	
18	Staff help me to set realistic goals.	0.618	
	**Engaging Authentically**	0.804	0.578
9	Staff connect with me as a person.	0.524	
11	When we disagree about my care, the staff try to find common ground.	0.558	
16	Staff listen to me and hear what I have to say about my care.	0.651	
	**Being Sympathetically Present**	0.734	0.479
2	Staff use my personal experiences to build a relationship with me.	0.473	
5	Staff give me their full attention when they are with me.	0.525	
14	Staff respond compassionately^a^ when I am upset or unhappy.	0.440	
	**Working Holistically**	0.844	0.575
4	Staff consider my home environment in meeting my care needs.	0.562	
8	Staff ask me about my life.	0.583	
13	I feel cared for.	0.529	
17	Staff understand my family circumstances when caring for me.	0.625	

Abbreviation: AVE, Average Variance Extracted.

^a^Compassion is the sensitivity shown by the healthcare professional to understand the suffering of another person, combined with the willingness to help and promote wellbeing in order to find a solution to the other's problem (Perez‐Bret et al. 2016).

## 4 Discussion

This study presents the PCPI‐C's first validated Spanish version, providing a culturally adapted and psychometrically sound instrument to assess PCP from the patient perspective. Adding this version to those already available in English, Portuguese, French and Danish strengthens the instrument's international applicability and supports cross‐cultural research based on a shared theoretical framework. Access to linguistically equivalent versions also facilitates the comparison of person‐centred practice across healthcare systems and enhances the generalisability of findings.

The PCPI‐C enables patients to express their perceptions of how PCP has been provided. Capturing this perception is not only essential to ensuring that patients truly experience PCP but also indicates the extent to which healthcare institutions effectively implement this approach. Because the PCPI‐C is rooted in the PCPF (McCormack et al. 2024) and not only in the literature's thematic analysis (Havana et al. 2023; Mitchell et al. 2022), the phenomenon it addresses is clearly defined and delineated. This foundational alignment ensures a transparent, unambiguous meaning of person‐centeredness for patients.

Evaluating content validity is fundamental to ensuring the quality of a translated instrument and serves as the basis for establishing further evidence of validity, such as construct validity derived from the target population's scores (Cook and Beckman 2006; Polit et al. 2007). In this research, the content validity analysis demonstrated that the PCP construct is adequately reflected across all 18 items. Notably, no item exhibited an inadequate value following adjustment via the weighted kappa index, which minimises the probability of chance agreement among expert opinions. This finding supports the appropriateness of the Spanish version and aligns with established recommendations for strengthening validation through both expert judgement and statistical analyses (Almanasreh et al. 2019).

Overall, the psychometric results confirm that the Spanish PCPI‐C preserves the theoretical coherence of the original instrument (McCormack et al. 2024, 2025), while demonstrating acceptable reliability and validity in a Spanish‐speaking context. These findings reinforce the instrument's suitability for assessing person‐centred processes and highlight the relevance of the Person‐centred Practice Framework as an organising structure for understanding patients' experiences across cultural contexts.

Despite adhering to recommended guidelines (McKown et al. 2020; Wild et al. 2005), subtle cultural or semantic factors may have influenced the performance of certain items. For example, the relatively low reliability of Construct 4 (*Being sympathetically present*; AVE = 0.479) and of Item 6 (‘I feel able to say to staff what is important to me’; item reliability = 0.381) may reflect such issues. Culturally, patients in some Spanish‐speaking settings may be less inclined to openly express personal concerns to healthcare staff. Semantically, the phrase ‘what is important to me’ may also lack precision in Spanish, where it can reference specific medical issues or broader personal values, such as family or emotional needs. This interpretive variability might lead to inconsistent responses and reduced measurement precision. These observations highlight the need to complement psychometric validation with qualitative approaches, such as cognitive interviewing (Willis 2005), to explore item interpretation in more depth.

Regarding structural validity, modifications to the original model were introduced cautiously and only when supported both theoretically and statistically. Nine correlated error terms were incorporated based on high modification indices (MI > 0.25), following an approach consistent with the original model (McCormack et al. 2024), which included six error covariances. These refinements improved model fit while preserving conceptual coherence and avoiding overfitting.

The refined model also provided insights into item relationships. For instance, the correlation between Items 6 and 7—initially reported as *r* = 0.45 (McCormack et al. 2024)—increased to *r* = 0.513 in this study. Both items belong to *Working with the person's beliefs and values*, reflecting the importance of feedback in reinforcing person‐centredness. As suggested by McCormack et al. (2024), the capacity to express what matters and to reflect on care received is central to building therapeutic relationships and co‐constructing meaning during care encounters. This strengthened correlation may also reflect the role of feedback in identifying care gaps and promoting quality improvement.

Another relevant correlation—between Items 1 and 2, not identified in the original validation but emerging here with moderate strength (*r* = 0.427)—appears to reflect shared decision‐making processes, where understanding patient priorities and integrating personal experiences are essential to meaningful care relationships. This aligns with Vareta et al. (2025), who emphasised the importance of integrating patient narratives into person‐centred processes and with the broader recognition of Patient‐Reported Experience Measures (PREMs) as indicators of healthcare quality.

A further correlation of interest was observed between Items 7 and 9 (*r* = 0.379), suggesting a link between expressing care experiences and perceiving relational depth. As discussed by Mabire et al. (2024), *engaging authentically* requires mutual recognition and emotional presence, supported by open and reciprocal communication. Feedback, in this context, becomes a relational act that strengthens the therapeutic alliance and fosters a sense of being known and valued.

Finally, the emergence of a negative correlation among error terms warrants theoretical reflection. Such correlations may indicate conceptual tension between items or cultural differences in how certain constructs are interpreted. As noted by Rosted et al. (2025), translation and cultural adaptation processes may uncover subtle shifts in meaning that influence item responses. This negative correlation likely reflects local conceptual divergences rather than a flaw in the theoretical model.

### 4.1 Limitations and Strengths

Several limitations should be acknowledged. First, although the translation and cultural adaptation process adhered to internationally recognised guidelines, some semantic ambiguity may persist. For example, the expression ‘what is important to me’ may be interpreted differently across Spanish‐speaking contexts, which could explain the lower reliability of Item 6. Second, although the sample size was adequate for psychometric testing, it may not fully represent the heterogeneity of Spanish‐speaking populations. Third, the cross‐sectional design limited the ability to assess test–retest reliability or responsiveness to change.

This study also has notable strengths. It provides the first validated Spanish version of the PCPI‐C, expanding access to a theoretically grounded measure of person‐centred care. The translation process adhered to rigorous international guidelines and incorporated feedback from both experts and patients, enhancing linguistic and conceptual equivalence. The psychometric evaluation used robust, theory‐driven CFA procedures and an adequate sample, resulting in a reliable and valid tool for Spanish‐speaking healthcare settings.

### 4.2 Future Implications

Future research should adopt longitudinal and intervention‐based designs to assess the Spanish PCPI‐C's sensitivity to changes over time, especially following person‐centred interventions. Additionally, qualitative studies employing cognitive interviewing techniques would provide deeper insights into how patients from diverse Spanish‐speaking cultures interpret key items, thereby improving the tool's semantic precision and cultural relevance.

From a professional practice perspective, the validated PCPI‐C offers clinicians, managers and researchers an acceptable instrument for evaluating the extent to which person‐centred principles are embedded in everyday care. The instrument's use may facilitate identification of gaps in care from the patient's perspective, track the evolution of PCP over time and evaluate the impact of organisational interventions designed to enhance PCP. At a broader level, this instrument can inform training and policy development, contribute to accreditation and quality assurance systems and support cross‐cultural benchmarking within and beyond Spanish‐speaking health systems. Future research should incorporate qualitative methodologies to explore cultural and semantic nuances further, thereby optimising the PCPI‐C's relevance, clarity and applicability across diverse contexts.

## 5 Conclusions

This study confirms that the PCPI‐C's Spanish version maintains the original instrument's theoretical integrity and acceptable psychometric properties, enabling the PCP's valid assessment from Spanish‐speaking patients' perspective. Observed conceptual convergence among certain items underscores the importance of integrating statistical and interpretive approaches in instruments' cross‐cultural adaptation. In a globalised world where major health challenges require placing people at the centre of any healthcare practice, this research is a crucial step for ensuring the development of better clinical outcomes and more ethical, inclusive and responsive models of care.

## Author Contributions

**Ana Choperena:** conceptualisation, methodology, data curation, resources, writing – original draft; **Ana Carvajal‐Valcárcel:** conceptualisation, methodology, formal analysis, writing – original draft, supervision, project administration, funding acquisition; **Mónica Vázquez‐Calatayud:** conceptualisation, methodology, data curation, resources, writing – original draft; **Juan Gamero‐Salinas:** methodology, formal analysis, writing – original draft. **Begoña Errasti‐Ibarrondo:** conceptualisation, data curation, writing – review and editing; **Virginia La Rosa‐Salas:** writing – review and editing, funding acquisition. **Brendan McCormack:** conceptualisation, writing – review and editing, supervision. **Vaibhav Tyagi:** supervision, writing – review and editing. **Marta Lizarbe‐Chocarro:** conceptualisation, methodology, formal analysis, data curation, writing – original draft, supervision, funding acquisition. All authors have read and agreed to the published version of the manuscript.

## Funding

This research received funding from the State Plan for I + D + I 2017–2020, with co‐financing provided by the ISCIII‐General Sub directorate for Research Evaluation and Promotion and the European Regional Development Fund (PI20/01644). The funding entity played no part in shaping the study's design, execution, data analysis, interpretation or manuscript composition.

## Conflicts of Interest

The authors declare no conflicts of interest.

## Supporting information


**Table S1:** Spanish version of the Person‐Centred Practice Inventory‐Care (PCPI‐C).

## Data Availability

The data that support the findings of this study are available from the corresponding author upon reasonable request.
