# Supplementary material for: Spanish Translation, Cultural Adaptation and Validation of the Person‐Centred Practice Inventory‐Care (PCPI‐C): Enhancing Collaborative Care and Patient Involvement
Source: J Adv Nurs. 2026 Feb 16;82(10):9159–72. doi: 10.1111/jan.70542 (PMC13576814; doi:10.1111/jan.70542)
Supplement: Supplementary file 1 [file JAN-82-9159-s001.docx]

**Table S1.** Spanish version of the Person-Centred Practice Inventory-Care (PCPI-C).

Piense y reflexione sobre el cuidado recibido a lo largo de su experiencia de salud y, por favour, indique el grado de acuerdo o desacuerdo (1*: Totalmente en desacuerdo. 2: En desacuerdo. 3: Neutral. 4: De acuerdo. 5: Totalmente de acuerdo*) con respecto a las siguientes afirmaciones.

| Ítem 1 | [El personal sanitario se esfuerza por comprender lo que es importante para mí](about:blank) |
| --- | --- |
| Ítem 2 | El personal tiene en cuenta mi experiencia personal para establecer una relación conmigo |
| Ítem 3 | El personal me involucra en la toma de decisiones acerca de mi cuidado |
| Ítem 4 | El personal tiene en cuenta mi entorno familiar para cubrir mis necesidades de cuidado |
| Ítem 5 | El personal me dedica toda su atención cuando recibo su cuidado |
| Ítem 6 | Me siento capaz de decir al personal sanitario lo que es importante para mí |
| Ítem 7 | Me siento capaz de transmitir al personal mi experiencia de cómo he sido cuidado |
| Ítem 8 | El personal se interesa por mi vida |
| Ítem 9 | El personal conecta conmigo como persona |
| Ítem 10 | El personal comprueba si tengo toda la información que necesito |
| Ítem 11 | Cuando no coincidimos en algún aspecto sobre mi cuidado, el personal intenta llegar a un acuerdo conmigo |
| Ítem 12 | El personal me cuida teniendo en cuenta el conocimiento que tiene sobre mí como persona |
| Ítem 13 | I siento cuidado/a |
| Ítem 14 | Cuando me siento disgustado o triste el personal sanitario me cuida de forma compasiva^1^ |
| Ítem 15 | El personal me ayuda a expresar mis preocupaciones sobre el tratamiento y el cuidado que recibo |
| Ítem 16 | El personal me escucha y tiene en cuenta mi opinión sobre el cuidado que recibo |
| Ítem 17 | El personal tiene en cuenta mis circunstancias familiares cuando me cuida |
| Ítem 18 | El personal me ayuda a establecer objetivos realistas |

^1^Compasión es la sensibilidad que muestra el profesional de la salud para comprender el sufrimiento de otra persona combinada con la voluntad de ayudar y de promover el bienestar con el fin de encontrar una solución a su problema (Pérez-Bret et al., 2016).
